# Diabetes mellitus and hypertension have comparable adverse effects on health-related quality of life

**DOI:** 10.1186/1471-2458-10-12

**Published:** 2010-01-13

**Authors:** Tamara Poljičanin, Dea Ajduković, Mario Šekerija, Mirjana Pibernik-Okanović, Željko Metelko, Gorka Vuletić Mavrinac

**Affiliations:** 1 Vuk Vrhovac University Clinic, Zagreb, Croatia; 2 Andrija Štampar School of Public Health, Medical School, University of Zagreb, Zagreb, Croatia

## Abstract

**Background:**

We aimed to assess health-related quality of life (HRQoL) among people with diabetes or hypertension, estimate the effect of cardiovascular comorbidities on HRQoL as well as compare HRQoL in these groups with that of healthy individuals.

**Methods:**

A total of 9,070 respondents aged 18 years and over were assessed for HRQoL. Data were obtained from the Croatian Adult Health Survey. Respondents were divided into five groups according to their medical history: participants with hypertension (RR), hypertension and cardiovascular comorbidities (RR+), diabetes mellitus (DM), diabetes and cardiovascular comorbidities (DM+) and participants free of these conditions (healthy individuals, HI). HRQoL was assessed on 8 dimensions of the SF-36 questionnaire.

**Results:**

Participants with diabetes and those with hypertension reported comparably limited (p > 0.05) HRQoL in all dimensions of SF-36, compared with healthy individuals (p < 0.05). If cardiovascular comorbidities were present, both participants with diabetes and participants with hypertension had lower results on all SF-36 scales (p > 0.05) than participants without such comorbidities (p < 0.05). The results remained after adjustment for sociodemographic variables (age, sex, employment, financial status and education).

**Conclusion:**

Diabetes and hypertension seem to comparably impair HRQoL. Cardiovascular comorbidities further reduce HRQoL in participants with both chronic conditions. Future research of interventions aimed at improving these participants' HRQoL is needed.

## Background

One of the most important goals of all health interventions is to improve the quality of life of persons affected by disease [1]. In the domain of physical health and illness, quality of life refers to participant's self-evaluation of health or on their perceived functional status and well-being [2]. In chronic conditions such as hypertension and diabetes, health-related quality of life (HRQoL) is an especially important outcome, given their lifelong nature and the need for daily self-management.

HRQoL has been found to be poorer in diabetic participants than in the general population [1,3], especially in the domains of self perceived physical health, while findings on domains of psychosocial functioning vary between studies [4-7]. Hypertension has also been shown to be associated with negative outcomes in HRQoL, especially in the domain of subjectively perceived general health, although its impact on HRQoL is usually less adverse than that of other chronic diseases [4,8].

Although studies comparing the effects of different chronic diseases generally point to a conclusion that hypertension has less adverse effects on HRQoL than diabetes, the magnitude and profile of differences between diabetic and hypertensive participants in HRQoL have not been determined as yet [9-11].

Epidemiological research of HRQoL in Croatia has so far been conducted only for the general population [12], while comprehensive research of HRQoL in Croatian participants with chronic conditions is still lacking. As diabetes and hypertension are among the chronic diseases that affect the largest number of individuals and lead to severe complications, the present study was aimed at determining HRQoL in diabetic and hypertensive participants, and to examining the effects of cardiovascular comorbidities on HRQoL in these chronic conditions.

## Methods

### Participants

This survey was a part of the Croatian Adult Health Survey (CAHS), a cross-sectional field survey conducted by trained health care professionals in 2003. Data collected in this study provided a comprehensive assessment of health of Croatian residents, including their HRQoL, access to and use of health care services, health status and determinants such as smoking, physical activity, nutrition and alcohol use. The study was approved by the Research Ethics Committee, Medical School, University of Zagreb, grant number 04-1060-2006. The design of the CAHS is described in more detail elsewhere [13].

### Sampling

A total of 9,070 respondents aged 18 years and over were assessed for HRQoL.

Respondents were divided into 5 groups: participants with hypertension (RR) (I10; ICD-10), participants with hypertension and cardiovascular co-morbidities (RR+), participants with diabetes mellitus (DM) (E10-E14; ICD-10), participants with diabetes and cardiovascular co-morbidities (DM+) and individuals without these conditions, i.e. healthy individuals (HI). Cardiovascular comorbidities were defined as self-reported history of angina (I20; ICD-10), myocardial infarction (I25.2; ICD-10), heart insufficiency (I50; ICD-10) or stroke (I61, I63, I64; ICD-10). Participants with both hypertension and diabetes were classified as DM+.

### Instruments

HRQoL was assessed by SF-36, the Medical Outcome Study short-form health survey [14-16]. The Croatian version of the SF-36 questionnaire was prepared using a standard translation procedure, followed by a pilot study carried out in 1998 [12]. Psychometric characteristics of this instrument have shown its adequate reliability and validity [17].

The questionnaire contains 36 items integrated in multi-item scales measuring eight generic health concepts: physical functioning (PF), social functioning (SF), role physical (RP), bodily pain (BP), mental health (MH), role emotional (RE), vitality (VT), and general health (GH).

Scoring included transformation of raw scores for each subscale to a 0-100 scale, with higher scores representing better quality of life.

### Statistical analysis

All statistical analyses were performed using SAS (version 9.1.3). Descriptive analysis included calculations of means, 95% confidence intervals (CI) of means and frequencies of categorical variables. Distributions of categorical sociodemographic predictors were compared using chi-square tests, with a downward adjustment of statistical significance for multiple comparisons. The corrected level of significance was obtained using Bonferroni correction, i.e. by dividing the defined p value (0.05) with the number of comparisons made (n = 10). The adjusted significant p value for multiple comparison of categorical predictors was thus less than 0.005 (p < 0.005). The level of significance of correlation between variables and the correlation trend were analysed by Pearson Test. The differences between groups and the impact of categorical sociodemographic variables were estimated by MANOVA. Continuous sociodemographic variables were controlled for by MANCOVA. All post-hoc comparisons were performed by Scheffe test. Statistical significance was defined as p value less than 0.05 (p < 0.05).

## Results

Participants' sociodemographic characteristics are presented in Table 1. There were statistically significant differences among different groups of participants and healthy individuals in age (p < 0.001), level of education, employment status and self-evaluation of financial status (p < 0.001). Healthy individuals were younger, better educated, more often employed and of better financial status than participants with hypertension and/or diabetes. Among participants with hypertension and/or diabetes, those with cardiovascular comorbidities (DM+ and RR+) were older, less educated, less often employed and of worse self-assessed financial status than participants without these comorbidities (DM and RR) (p < 0.001). The only insignificant differences were those for self-assessed financial status among participants with diabetes compared with participants who had cardiovascular comorbidities (DM vs DM+ and DM vs RR+). Participants with a matching status of comorbidities (RR vs DM and RR+ vs DM+) did not differ in sociodemographic predictors. All groups consisted of more women than men. There was a statistically significant difference in sex (p < 0.001) between the groups without complications (DM and RR) with predomination of women with hypertension, while the difference was insignificant (p = 0.073) between the groups with complications (DM+ and RR+).

**Table 1:** Sociodemographic characteristics of the studied groups

		HI (N = 5.043)	RR (N = 1.490)	RR+ (N = 1.564)	DM (N = 231)	DM+ (N = 742)	p	significant differences^b^
Age^a^		46.5 (46.1-47.0)	60.1 (59.4-60.8)	65.3 (64.6-65.9)	60.0 (58.4-61.7)	65.7 (64.9-66.5)	< 0.001	RR&RR+, RR&DM+, DM&DM+, DM&RR+, HI&all other groups

Gender	Male	32.8%	29.9%	28.7%	44.6%	32.3%	< 0.001	DM&DM+, DM&RR+, RR &DM, RR+&HI, DM&HI
			
	Female	67.2%	70.1%	71.3%	55.4%	67.7%		

Level of education	Unfinished primary school	9.8%	24.2%	31,1%	16.0%	30.7%	< 0.001	RR&RR+, RR&DM+, DM&DM+, DM&RR+, HI&all other groups
			
	Primary school	20.1%	26.9%	28.8%	28.6%	27.8%		
			
	High school or similar	53.5%	37.4%	31.2%	43.3%	32.4%		
			
	College or University	16.6%	11.5%	8,9%	12.1%	9.1%		

Employed	Yes	44.0%	22.3%	11.9%	21.2%	10.4%	< 0.001	RR&RR+, RR&DM+, DM&DM+, DM&RR+, HI&all other groups
			
	No	56.0%	77.7%	88.1%	78.8%	89.6%		

Self-evaluation of financial status	Poorer than average	36.8%	45.0%	54.7%	45.4%	53.0%	< 0.001	RR&RR+, RR&DM+, RR&HI, RR+&HI, DM+&HI
			
	Average	50.2%	45.5%	39.3%	45.9%	40.4%		
			
	Better than average	13.0%	9.5%	6.0%	8.7%	6.6%		

Data are presented as proportion (%) unless noted otherwise; ^a ^Mean (± 95% confidence interval) ^b ^Pairs with significant differences between groups; post-hoc analysis with downward adjustment for multiple comparison; Abbreviations: HI - healthy individuals, RR - participants with hypertension, RR+ - hypertension and other cardiovascular comorbidities, DM - diabetes mellitus, DM+ - diabetes mellitus and cardiovascular comorbidities

Data on HRQoL are presented in Table 2. All dimensions of SF-36 were significantly lower among participants with diabetes and/or hypertension than among healthy individuals (p < 0.001). Groups with complications (DM+ and RR+) had poorer HRQoL on all dimensions of SF-36 in comparison with the healthy individuals (p < 0.001) as well as those without complications (DM and RR) (p < 0.05).

**Table 2:** The unadjusted and adjusted HRQoL indicators in the studied groups.

	HI (N = 5,043)	RR (N = 1,490)	RR+ (N = 1,564)	DM (N = 231)	DM+ (N = 742)	p^a^
PF	81.5 (80.9-82.2)	66.6 (65.2-68.0)	47.8 (46.3-49.2)	65.1 (61.5-68.7)	49.9 (47.8-52.0)	< 0.001
	*78.1 (76.8-79.5)*	*67.8 (66.0-69.7)*	*51.7 (49.7-53.7)*	*69.4 (65.4-73.4)*	*57.3 (54.6-60.0)*	

RP	77.5 (76.4-78.5)	58.1 (55.9-60.3)	33.5 (31.4-35.6)	56.1 (50.2-61.9)	35.9 (32.7-39.1)	< 0.001
	*73.3 (71.1-75.5)*	*60.3 (57.3-63.3)*	*38.1 (34.8-41.3)*	*61.1 (54.6-67.7)*	*43.4 (39.0-47.8)*	

BP	74.5 (73.7-75.3)	62.1 (60.7-63.6)	48.5 (47.1-50.0)	58.7 (54.9-62.5)	49.4 (47.2-51.6)	< 0.001
	*72.8 (71.2-74.3)*	*65.5 (63.4-67.6)*	*52.7 (50.3-57.0)*	*62.9 (58.3-67.6)*	*54.0 (50.9-57.1)*	

GH	64.2 (63.6-64.8)	51.6 (50.6-52.6)	40.2 (39.2-41.1)	49.0 (46.3-51.7)	39.2 (37.8-40.6)	< 0.001
	*62.9 (61.8-64.0)*	*53.2 (51.7-54.7)*	*43.7 (42.1-45.3)*	*52.7 (49.5-55.9)*	*41.7 (39.6-43.9)*	

VT	60.7 (60.1-61.2)	53.2 (52.2-54.2)	39.8 (38.7-40.9)	51.4 (48.5-54.3)	41.3 (39.7-43.0)	< 0.001
	*59.1 (57.9-60.2)*	*55.4 (53.9-57.0)*	*44.4 (42.7-46.1)*	*55.0 (51.7-58.4)*	*46.5 (44.2-48.7)*	

SF	82.0 (81.4-82.7)	74.4 (73.1-75.6)	58.7 (57.2-60.2)	69.2 (65.6-72.8)	59.8 (57.6-61.9)	< 0.001
	*79.2 (77.8-80.6)*	*76.1 (74.2-78.0)*	*62.7 (60.6-64.8)*	*73.9 (69.8-78.1)*	*64.7 (61.9-67.4)*	

RE	79.9 (78.9-80.9)	69.3 (67.1-71.4)	51.4 (49.1-53.7)	70.4 (64.9-76.0)	51.5 (48.1-54.9)	< 0.001
	*76.6 (74.3-78.8)*	*72.6 (69.5-75.7)*	*57.1 (53.7-60.5)*	*74.7 (67.9-81.4)*	*58.8 (54.2-63.3)*	

MH	68.3 (67.8-68.8)	62.5 (61.5-63.4)	52.8 (51.7-53.8)	60.6 (58.1-63.2)	53.7 (52.1-55.3)	< 0.001
	*66.8 (65.7-67.8)*	*64.0 (62.6-65.5)*	*57.8 (56.2-59.4)*	*63.4 (60.2-66.5)*	*58.1 (56.0-60.2)*	

Data are presented as mean (± 95% confidence interval); adjusted results are in italic format; ^a ^post-hoc analysis showed significant differences between RR&RR+, RR&DM+, DM&DM+, DM&RR+ and HI& all other groups (RR, RR+, DM, DM+) in all SF-36 dimensions; Scheffe test, p < 0.001; Abbreviations: HI - healthy individuals, RR - participants with hypertension, RR+ - hypertension and other cardiovascular comorbidities, DM - diabetes mellitus, DM+ - diabetes mellitus and cardiovascular comorbidities, PF - physical functioning, RP - role physical, BP - bodily pain, GH - general health, VT - vitality, SF - social functioning, RE - role emotional, MH - mental health.

The studied groups differed significantly in all dimensions of SF-36 (p < 0.001). In post-hoc analyses we found that, regardless of diabetes or hypertension status, participants with comorbidities had comparable, significantly poorer HRQoL than participants without comorbidities (p < 0.001). Age showed small to medium correlations with all dimensions of HRQoL (p < 0.05); when controlled for, differences between the groups of participants with (DM+ and RR+) and those without comorbidities (DM and RR) remained significant (p < 0.001), as well as differences between healthy individuals (HI) and all other groups (DM, DM+, RR, RR+). Adjusted data on HRQoL are also presented in Table 2. Unemployment, lower self-evaluated financial status and lower level of education were associated with worse self-assessments of HRQoL in all subgroups of participants (p < 0.05).

Women had consistently lower scores on all dimensions of SF-36 (p < 0.001), indicating poorer HRQoL. In the groups without complications (DM and RR), stratified analysis according to sex revealed poorer social functioning and more bodily pain (p < 0.05) in women with diabetes (DM) than in women with hypertension (RR), while all other differences were insignificant (p > 0.05). There were no significant differences between diabetic (DM) and hypertensive (RR) men in any of the SF components.

All differences in HRQoL between participants with and without comorbidities were clinically relevant, as they were greater than 5 points on a 0-100 scale which is found to be clinically significant [18]. Impairments in HRQoL dimensions of participants with comorbidities were most pronounced in the domain of physical roles (RP), social functioning (SF) and physical functioning (PF).

## Discussion

Our results suggest that diabetes and hypertension have comparably negative effects on HRQoL, which supports the conclusion that both conditions impose similar self perceived limitations in physical and mental functioning. However, available literature [1] suggests that diabetes in its early stages has no impact on perceived HRQoL of the affected individuals, while our study found lower scores on the majority of SF-36 dimensions, even in participants without cardiovascular comorbidities. This might suggest that assessing HRQoL may be beneficial even in the early stages of treatment for diabetes and hypertension.

In our study all dimensions of HRQoL were poorer in people with diabetes and hypertension (DM and RR), as well as in participants with cardiovascular complications (DM+ and RR+), compared with healthy individuals (HI). However, our data did not include information on possible non-vascular comorbidities, such as retinopathy, nephropathy or musculoskeletal disease, which have been found to be important predictors of HRQoL in diabetes [1,2,5]. It is likely that some individuals who had been classified into the groups without cardiovascular comorbidities of hypertension or diabetes had other comorbidities that potentially reduced their HRQoL. On the other hand, even in the absence of any comorbidities, a mere perception of being chronically ill and thus frailer than a healthy person may also detrimentally affect participant's HRQoL [19,20].

We also observed that cardiovascular comorbidities had a particularly negative impact on HRQoL in these two chronic diseases. This is in line with previous research which has found that the presence of macrovascular complications was a very important factor that comparably limited participants' HRQoL [1,2] to a clinically significant extent [18] in both hypertension and diabetes. Cardiovascular comorbidities are thought to debilitate physical functioning to a higher degree than hypertension or diabetes alone. This can be substantiated by profiles of particular SF-36 scales, as self-reported well-being in participants with comorbidities has been shown to be poorest with respect to physical functioning (RP and PF).

Previous studies have reported that diabetes and hypertension negatively affect participants' HRQoL [4,5,21], and that heart disease worsened these participants' HRQoL even further [6,22-27]. However, other studies have yielded conflicting results on the severity of impact of diabetes and hypertension on HRQoL [28,29], without demonstrating whether the degree of impact of cardiovascular comorbidities in these two conditions is comparable.

Between-group comparisons of demographic characteristics (level of education, employment status and self-evaluation of financial status) in paired participant groups (DM vs RR and DM+ vs RR+) did not reveal statistically significant differences. Although these demographic factors influence HRQoL, their effect may be presumed to have been comparable in the groups with complications (DM+ and RR+), as well as in those without complications (DM and RR). Taking into account the differences in sex between the groups without complications (DM and RR), we analysed both sexes separately and found differences in social functioning and bodily pain, but only in women. In view of these results and of the fact that HRQoL was similarly affected in the pairs of groups with complications (DM+ and RR+) and in those without them (DM and RR) even after age was controlled for, we conclude that the observed differences are attributable to chronic conditions rather than to possible sociodemographic confounders analysed in this study.

Our results confirm previous reports of adverse impacts of diabetes and hypertension on participants' well-being and functioning. The novelty of our study is that it shows these effect sizes are comparable. This is a useful finding for clinical practice, particularly in the early treatment of diabetes and hypertension, at which point improvement in self-management and consequently HRQoL is still possible. Our results also contribute to transcultural research of HRQoL in diabetes and hypertension, as research on this issue in transitional countries has been scarce.

Limitations of our study include the self-reported nature of the CAHS data, and that more detailed information on disease duration, adherence to treatment, and non-vascular concomitant diseases were unavailable. However, all data were collected by trained medical professional, which could provide some certainty that medical conditions studied were recognized and classified properly.

## Conclusions

Our results showed an adverse impact of both diabetes and hypertension on participants' well-being and functioning, adding to current knowledge by the finding that the effect sizes are comparable, at least in the Croatian adult population. This could be useful in clinical practice, particularly in early treatment of diabetes and hypertension, at which point improving self-management and consequently HRQoL is still possible. We also found that cardiovascular comorbidities have a marked negative impact on HRQoL in people with both these conditions.

Early recognition of participants with these chronic diseases might therefore be considered to be of importance, not only in clinical disease management, but also in participant education and empowerment aimed at improving their HRQoL. Further research on appropriate interventions aimed at HRQoL of these participants is needed.

## Abbreviations

HRQoL: health-related quality of life; DM: diabetes mellitus; DM+: diabetes mellitus and cardiovascular comorbidities; RR: hypertension; RR+: hypertension and other cardiovascular comorbidities; PF: physical functioning; SF: social functioning; RP: role physical; BP: bodily pain; MH: mental health; RE: role emotional; VT: vitality; GH: general health

## Competing interests

The authors declare that they have no competing interests.

## Authors' contributions

TP participated in study design, drafted the manuscript and performed the statistical analysis. DA and MS prepared the data for statistical analysis and participated in the preparation of the manuscript. MPO and ZM participated in study design, and revised the manuscript critically. GVM was included in conduction of Croatian Adult Health Survey (CAHS) and participated in study design. All authors have read and approved the final version of the manuscript.

## Pre-publication history

The pre-publication history for this paper can be accessed here:

http://www.biomedcentral.com/1471-2458/10/12/prepub
